# Recent Study Advances in Flexible Sensors Based on Polyimides

**DOI:** 10.3390/s23249743

**Published:** 2023-12-10

**Authors:** Tianyong Zhang, Yamei Chai, Suisui Wang, Jianing Yu, Shuang Jiang, Wenxuan Zhu, Zihao Fang, Bin Li

**Affiliations:** 1 Tianjin Key Laboratory of Applied Catalysis Science and Technology, School of Chemical Engineering and Technology, Tianjin University, Tianjin 300354, China; tyzhang@tju.edu.cn (T.Z.); yameichai@tju.edu.cn (Y.C.); wangsuisui@tju.edu.cn (S.W.); yjn0237_@tju.edu.cn (J.Y.); shuangjiang@tju.edu.cn (S.J.); 3020207329@tju.edu.cn (W.Z.); 3020207318@tju.edu.cn (Z.F.); 2 Collaborative Innovation Center of Chemical Science and Engineering (Tianjin), Tianjin 300072, China; 3 Tianjin Engineering Research Center of Functional Fine Chemicals, Tianjin 300354, China

**Keywords:** polyimide, flexible substrate, photosensitive polyimide, colorless polyimide, sensing, electrospinning, triboelectric nanogenerator, laser-induced graphene, fiber Bragg grating

## Abstract

With the demand for healthy life and the great advancement of flexible electronics, flexible sensors are playing an irreplaceably important role in healthcare monitoring, wearable devices, clinic treatment, and so on. In particular, the design and application of polyimide (PI)-based sensors are emerging swiftly. However, the tremendous potential of PI in sensors is not deeply understood. This review focuses on recent studies in advanced applications of PI in flexible sensors, including PI nanofibers prepared by electrospinning as flexible substrates, PI aerogels as friction layers in triboelectric nanogenerator (TENG), PI films as sensitive layers based on fiber Bragg grating (FBG) in relative humidity (RH) sensors, photosensitive PI (PSPI) as sacrificial layers, and more. The simple laser-induced graphene (LIG) technique is also introduced in the application of PI graphitization to graphene. Finally, the prospect of PIs in the field of electronics is proposed in the review.

## 1. Introduction

Within the emerging field of flexible electronics, intelligent sensors, integrating both semiconductor devices and integrated technology, have been developing towards flexibility. Compared with traditional sensors with the drawback of rigidity, flexible sensors have the characteristics of low cost, wearability, light weight, and simple structures, which enable them to catch target analytes much more effectively and obtain greater quality signals [1]. In addition, flexible sensors, maintaining high sensitivity and stretchability, are also being applied in many emerging fields (e.g., the biomedical field, intelligent transportation, wearable electronics, smart homes, etc.) [2]. Recently, Bao Z. N., Rogers A. J., Someya T., and other researchers in the domain of flexible sensors have proposed a technology roadmap of flexible sensors, mainly focusing on the issue of compatible sensor biological interface [3].

The strategy design, material selection, and fabrication method of flexible sensors are absolutely vital to promote the sensitivity and reliability of flexible devices. The materials used for flexible sensors include substrate materials, active materials, and flexible electrodes [4]. Polymers with the combination of easy preparation process, good chemical stability, and high mechanical compliance are ideal materials for making flexible sensors. Commonly used polymers include polyimide (PI) [5], poly(dimethylsiloxane) (PDMS) [6,7], poly(ether-ether-ketone) (PEEK) [8], polycarbonate (PC) [9], polyethylene naphthalate (PEN) [10], poly (ethylene terephthalate) (PET) [11] and polyurethane (PU) [12,13], and polyvinylidene fluoride (PVDF) [14,15,16,17]. Among these polymers, PI is extensively used owing to its excellent thermal stability [18], high chemical resistance, good dielectric properties, outstanding mechanical strength, and other comprehensive properties. However, PI is commonly not colorless and is unable to recover under great strain; thus, its application in the field of transparent flexible substrates is limited. Colorless polyimide (CPI) films have been achieved, which make the production of high-performance sensor devices possible [5,19]. In addition to the commonly used PI films, PI foams, PI fibers, PI aerogels, and so on have also been prepared. Above all, porous PI aerogels and PI fibers are used as the tribo-contact layer of triboelectric nanogenerators (TENG) and can greatly improve the performance of TENG [20,21,22]. On account of the triboelectrification and energy harvesting (EH) benefits of TENG, it can be used for the testing of embedded applications, especially those involving self-powered sensors [23]. Last but not least, with the emergence of three-dimensional (3D) printing and other technologies, there is a growing amount of research on developing electronic components directly on flexible substrates, which undoubtedly widens the application range of polymers [24].

**Figure 1:**
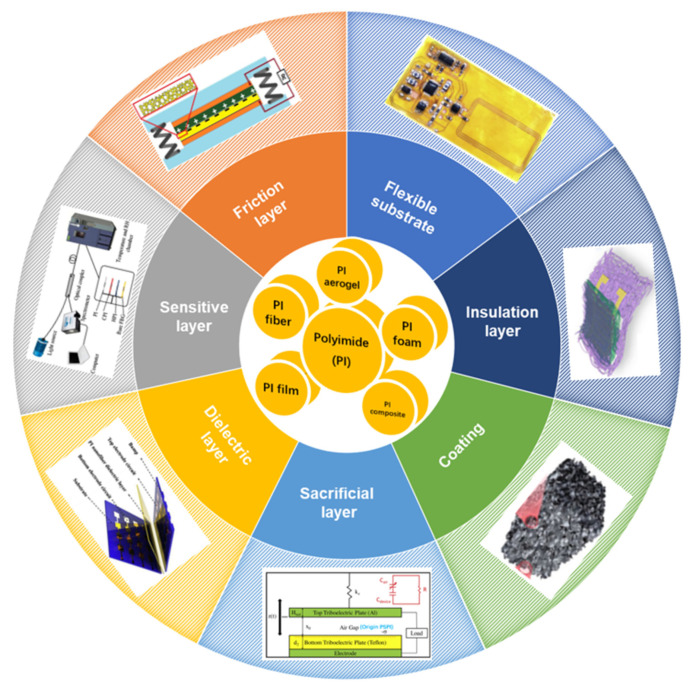
The applications of PI in flexible sensors.

Herein, this review explores flexible electronic materials in the application of sensors and summarizes the recent research and application of PIs in flexible sensors, including PI nanofibers prepared by electrospinning as flexible substrates, PI aerogels as tribo-contact layers in TENG, PI films as sensitive layers, insulation layers, sacrificial layers, and coatings (Figure 1). In particular, the application of PI graphitization to graphene by laser-induced graphene (LIG) technique without the need for superhigh temperature in sensors is introduced. Finally, this study proposes an orientation of future PI modifications and perspectives on the challenges of PI applications in the field of flexible electronics.

## 2. Applications of PI in Sensors

### 2.1. Flexible Substrate

#### 2.1.1. Traditional Flexible Substrate

**Figure 2:**
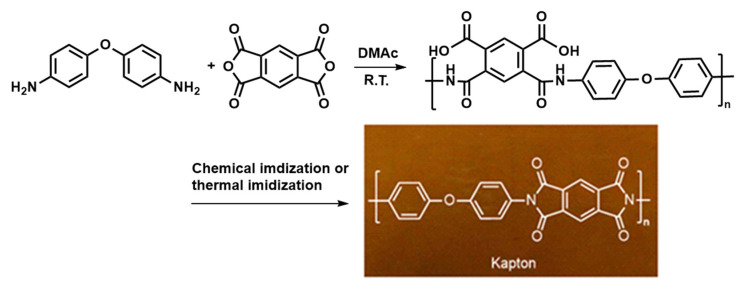
Synthesis diagram of Kapton.

Flexible electronic sensors featuring lightweight, flexible, and foldable characteristics have become an enthusiastic topic in the electronics field in recent years. Recent progress has been made in the research and application of flexible organic light-emitting diodes (OLED), flexible solar panels, flexible integrated circuits (IC), electronic skin (e-skin) [25], implantable medical and wearable devices [26], and so on. The rapid progress of flexible electronic devices depends on the use of new materials and the introduction of new manufacturing methods. Among them, PI is the most widely used flexible substrate by virtue of its excellent heat resistance, chemical resistance, and mechanical strength, and its ability to match the traditional semiconductor manufacturing process. Kapton, a product developed by DuPont, is a kind of PI with the highest utilization rate as a flexible substrate in traditional flexible pressure sensors. Figure 2 shows the synthesis process of Kapton with dark brown color from pyromellitic dianhydride (PMDA) and 4,4′-diaminodiphenyl ether (ODA) in polar solvent N,N-dimethylacetamide (DMAc). The method of preparing PI by forming polyamic acid (PAA) solution and then dehydration through chemical imidization or thermal imidization is known as two-step. The flexible sensors based on PI can be classified according to sensing principles or applications. Since there have been a large number of literature reviews in this field [1,2,19], only a few representative examples of PIs used as flexible substrates in flexible sensors are introduced here.

**Figure 3:**
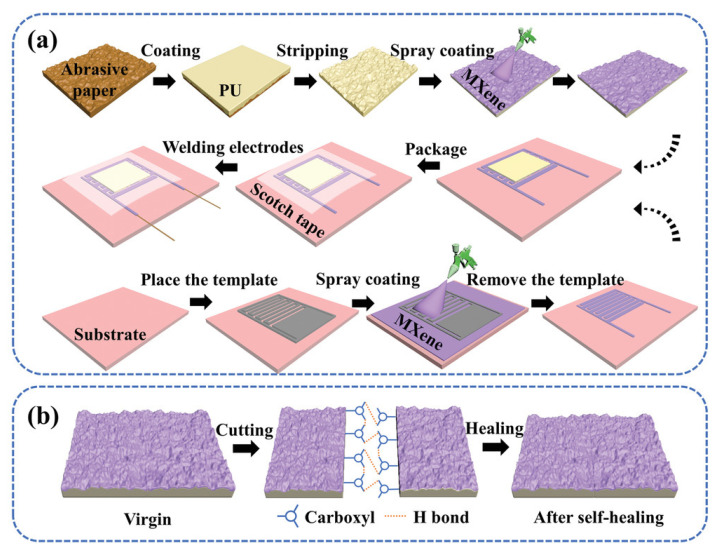
(**a**) Fabrication procedure of MXene-based sensor; (**b**) the self-healing mechanism of the sensitive layer [27]. Copyright 2022, John Wiley and Sons.

Yang et al. [27] fabricated a flexible piezoresistive sensor that is applied to e-skin and a manipulator with superior performance based on MXene/PU/interdigital electrodes (Figure 3). Similar to graphene, MXene is composed of two-dimensional transition metal carbides and nitrides with excellent hydrophilicity, good conductivity, large specific capacitance, and superior electrochemical performance. It has been one of the most popular conductive materials in flexible sensors recently [28,29,30]. Interestingly, the sensitive layer of the sensor uses PU with a self-healing ability as the substrate to improve the robustness of the system. The self-healing function takes advantage of the interaction between hydrogen bonds of PU. It is worth mentioning that the preparation of polymer materials with self-healing function is also a challenge in the sensing field [31,32,33,34], and PI with self-healing function will be mentioned in Section 2.2. The choice of flexible substrate is generally restricted by the fabrication processes of flexible sensors, but it is worthwhile mentioning that a versatile, low-cost, and universal template spraying method is used to prepare the interdigital electrodes here. MXene is separately sprayed on the spinosum structure PU as a sensitive layer and on the flexible substrates as an interdigital electrode. The selection of a flexible substrate for the interdigital electrode fabricated by this procedure can theoretically be random [27]. PI and a mixed cellulose filter membrane are selected, and experimental results are gained. There are differences in the performance of pressure sensors made with the two different flexible substrates above, and PI substrates obviously perform better. In comparison to cellulose filter membrane, the response time and the recovery time of the PI-based sensor are shorter, and its sensitivity is higher in low (0.20–1.70 kPa), middle (1.70–5.70 kPa), and high (5.70–20.30 kPa) pressure as well. In particular, the sensitivity of the sensor can reach 509.78 kPa^−1^ when it is within the middle-pressure range. The sensing performance comparison results are shown in Table 1. These results prove that the microstructural design of the flexible substrate does have an impact on the sensitivity property of the sensor. The results show that the PI-based sensor has better performance, and the fabrication process of this sensor is valuable, as it broadens the selection of flexible substrates and makes sense for the production of flexible sensors.

#### 2.1.2. PI Nanofiber Prepared by Electrospinning

With the improvement of sensor manufacturing technology, the advantages of traditional PI film (like Kapton) as a flexible substrate are less obvious. Furthermore, PI film with poor air permeability is not appropriate for long-time wearing, such that it is almost excluded from the application of sensors in human healthcare monitoring and wearable electronics [35]. Therefore, novel preparation processes and new structures of PI are constantly being explored. Converting PI into PI fiber to improve biocompatibility could be considered. Electrospinning technology is an effective and convenient method for preparing continuous nanofibers. Generally, PAA solution is synthesized first, and then PAA nanofibers are prepared by spinning. PI nanofibers are obtained through thermal or chemical imidization. For some soluble PIs, a one-step synthesis of PI solution can be used to directly prepare PI nanofibers through electrospinning [36,37,38]. The work of combining photosensitive polyimide (PSPI) and electrospinning to achieve a fiber-based photolithography hierarchical structure and micron-size patterns on flexible substrate will be introduced in detail below.

**Figure 4:**
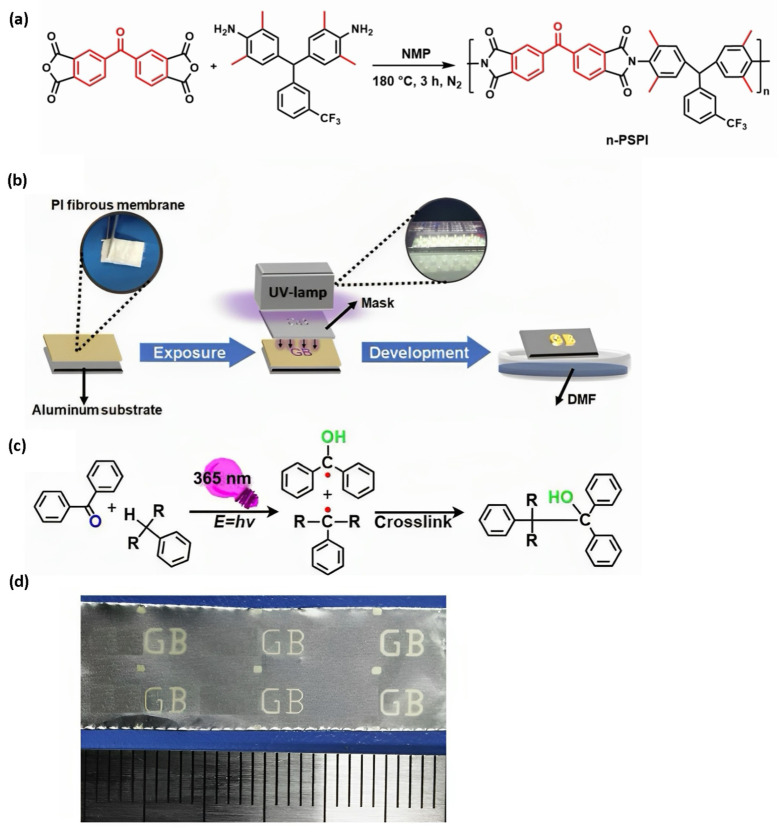
(**a**) Synthesis diagram of n-PSPI; (**b**) lithography process of PI UFMs; (**c**) mechanism of photo crosslinking reaction of PI fibers; (**d**) micropatterns gained by PI UFMs [39]. Copyright 2021, American Chemical Society.

A kind of ultrafine fibrous membrane (UFM) was fabricated by using a high-speed electrospinning technique and negative PSPI (n-PSPI) with a structure of aryl ketones, a photoactive group [39]. Although high-performance PI fiber has a mature synthesis process, the research on the combination of PSPI and electrospinning is lacking. The n-PSPI is synthesized via a one-step method from 3,3′,4,4′-benzophenonetetracarboxylic dianhydride (BTDA) and 4,4′-((3-(trifluoromethyl)phenyl)methylene)bis(2,6-dimethylaniline) (Figure 4a) [40]. The mechanism for preparing n-PSPI is to use the hydrogen reaction between the electrophilic carbonyl group of benzophenone and the alkyl hydrogen donor in the diamine unit under ultraviolet (UV) light (365 nm) to crosslink, which is insoluble in the developer and displays the micron-size pattern (Figure 4c). It is worth noting that the diamine molecule is elaborately designed. The methyl groups on the ortho position of amino use the steric effects to improve the glass transition temperature and solubility of PI. Trifluoromethyl can improve the optical transparency and reduce the moisture absorption and dielectric constant of PI. The well-designed diamine molecule and BTDA with benzophenone structure can produce photo cross-linking with high efficiency without other additives. In addition, electrospun PI fibers can provide outstanding mechanical properties and tailored physicochemical properties, so they are promising as a flexible substrate for the production of flexible electronics [41]. More essentially, a quantity of sophisticated patterns on the soft PI substrate can improve the mechanical tolerance of devices, so it is important to make fibers form hierarchical structures with various patterns at the micro- or nano-level through photolithographic approaches to obtain upgraded sensing properties and a variety of functions. In general, a layer of photoresist needs to be coated on the PI surface and etched by a lithography machine to realize patterning on PI. However, the photolithography machine is expensive, and the process is complex. In contrast, the PSPI, a more economical and practical way to realize patterning under UV irradiation, has been widely studied. The n-PSPI with good organic solubility by high-speed rotating (speed is 1000, 1500, 2000, and 2500 rpm) can form aligned ultrafine fibers with excellent heat resistance and lithography eligibility. Furthermore, PI UFMs can achieve micron-scale patterns by an easy process (Figure 4b,d) and maintain the fibrous structure. These phenomena are extremely important for future flexible electronic devices with complicated multilevel structures and functionality. The n-PSPI is also an important direction in the development of PIs, which can be patterned under light by introducing photosensitive groups and crosslinking agents into the PI system. At present, PSPI has been used as stress-buffer layers, redistribution layers, and protective layers for electronic packaging of IC. In order to adapt the development requirements of sensors and other electronic fields, how to decrease the curing temperature of PSPI is an urgent research topic [42].

### 2.2. Negative Friction Layer in TENG

Although TENG technology, depending on electrostatic induction to convert mechanical energy into electrical energy, is not the latest one in sensors, it has still become an increasingly attractive solution for self-powered sensors [43]. Owing to the principle of TENG, the surface properties of tribo-contact layers play a vital role in the output performance of TENG. Because the structure of PI contains a large number of imide groups, the electron cloud density is high, and it is easy to attract the surrounding positive charge to make the membrane surface negatively charged. In addition to splendid triboelectric negativity, PI has good thermal stability, so it is often used as a component for the preparation of TENG. The following will give examples of improving the properties of TENG by using different PI preparation processes and the structural design of PI.

#### 2.2.1. PI Aerogel as Friction Layer

**Figure 5:**
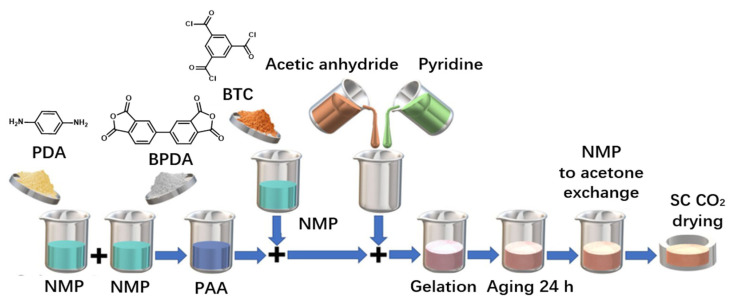
Preparation of PI aerogel [21]. Copyright 2019, Springer Nature.

Considering the thickness, mass, and charge storage capacity of the friction layer, PI aerogel was creatively proposed as a friction layer and compared with a compressed PI layer [20]. PI aerogel has countless nano-sized pores, which makes it have larger specific surface area and reduces the effective dielectric thickness of the layer, so that more charges are generated in the process of triboelectrification, ultimately leading to an increase in the capacitance of TENG. They also obtained samples with different ratios of open-cell contents by compressing aerogels at different rates [21]. The results show TENG prepared by PI aerogel with 50% pore content has the best electrical output performance. Compared with compressed PI, the open circuit voltage (V_oc_) is increased from 10 V to 40 V, the short circuit current (I_sc_) is increased from 2.4 μA to 5 μA, and the maximum instantaneous power (P_t max_) can reach 47 μW (the best resistance is 10 MΩ). When the open-cell content increases constantly, the performance of TENG descends, because the dielectric constant of air is less than that of PI. It can be explained as follows: when the open-cell content is excessive, the effective dielectric constant of the material will be reduced, and then the performance of Teng shows an increase. However, all PI-aerogel-based TENGs have better performance than the compressed-PI-based TENG. Figure 5 shows the preparation process of PI aerogel, in which 4-Phenylenediamine (PDA) and 3,3′,4,4′-biphenyl tetracarboxylic dianhydride (BPDA) are selected as monomers to react in N-methylpyrrolidone (NMP) (47 wt% solid content) to form PAA solution. Then, 1,3,5-Benzotriacyl chloride (BTC) is added as a crosslinker to improve the dielectric properties of PI. Acetic anhydride and pyridine are added as the dehydration agent and catalyst, respectively, to form PI through chemical imidization. Acetone is used for solvent exchange with NMP, and finally dried with supercritical carbon dioxide (SC CO_2_). Here, the method of preparing PI aerogel is conventional, but the innovative point of this work is to think of using aerogel instead of an ordinary PI film as the friction layer, which expands the application of PI aerogel. This is of great significance for the performance improvement of TENG and the application of PI aerogel.

#### 2.2.2. Modified PI as Friction Layer

**Figure 6:**
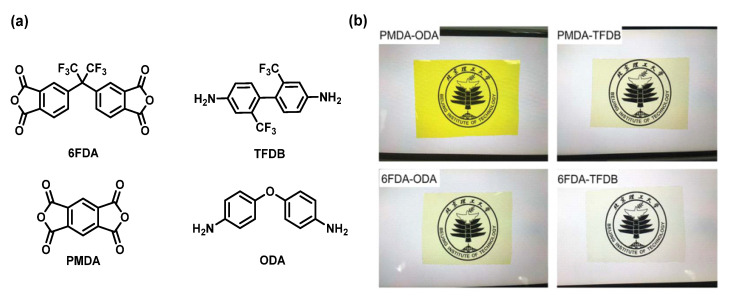
(**a**) Monomer structure of PIs; (**b**) transparency of prepared PI films [44]. Copyright 2021, Royal Society of Chemistry.

To use TENG for mobile screen display, the friction layer needs to be colorless. Wu et al. [44] synthesized a transparent-PI-based TENG for mobile phone screen. Four kinds of PI films are synthesized by combining two kinds of diamine (2,2′-bis(trifluoromethyl) benzidine (TFDB) and ODA) and two kinds of dianhydride (PMDA and 4,4′-(hexafluoroisopropylidene)diphthalic anhydride (6FDA)) in a conventional two-step process (Figure 6). It is found that 6FDA-TFDB film has excellent transparency. In fact, this phenomenon is well explained from the perspective of its molecular structure. The trifluoromethyl group is an electron-withdrawing group and conducive to the electrical output of TENG, which can effectively decrease the density of the electron cloud and improve the polarity of PI. In addition, a lot of work shows that fluorinated PI can endow PI with good optical properties without decreasing its thermal stability [5]. The friction coefficient in the friction process is also tested. It is found that the friction coefficient of fluorinated PI is less than that of Kapton, which could improve the durability of TENG [44]. Due to the strong electronegativity and small atomic radius of the fluorine atom (F), the electron and ion polarizability of PI containing F in some special position can be significantly reduced, thus reducing the dielectric constant of the PI. In addition, the introduction of F reduces the regularity of PI molecular chains, making the stacking of polymer chains more irregular, increasing the intermolecular space and further reducing the dielectric constant. However, PI with low dielectric constant cannot achieve high power output as a friction layer [45,46], so use of fluorinated transparent PI as a friction layer is not optimal. Regardless, colorless is a necessary condition for the special application of electronic display screens, so the preparation of a colorless, high-dielectric-constant, and low-dielectric-loss PI or PI composite can be a research direction in the future. 

**Figure 7:**
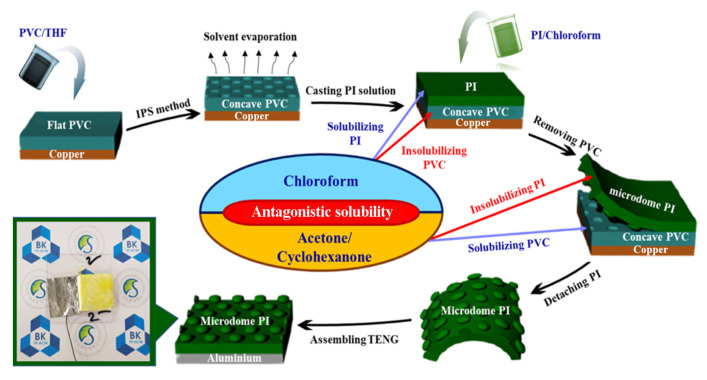
The fabrication process of TENG assembling md-PI [47]. Copyright 2022, Elsevier.

In addition to the monomer structure design mentioned above, changing the surface morphology by solubility is also an effective strategy to modify PI. Bui et al. [47] took advantage of the solubility difference of PI in solvent and non-solvent and realized customizable non-tightly-packed micro dome arrays on the PI surface (md-PI), which improved the effective contact area and contact pressure of the surface. The md-PI can be assembled in TENG and used under high-temperature (below 200 °C) and high-humidity conditions with durability and excellent electric output. The fabrication procedure is shown in Figure 7. Although the improved phase separation (ISP) method is simple and economical, it has strict requirements for solubility. Here, PI is required to be soluble in chloroform, but insoluble in the mixed solvent of acetone and cyclohexanone. The commonly used Kapton and unmodified PI are usually insoluble in low-boiling organic solvents (e.g., tetrahydrofuran (THF), chloroform, acetone, dichloromethane, cyclopentanone, etc.). The solubility of PI mainly depends on the chemical structure of the polymer. The strategy of designing soluble and processable PI is to reduce the rigidity or symmetry of the backbone, on the one hand, and to minimize the density of the imide ring along the skeleton, on the other hand [48]. 

**Figure 8:**
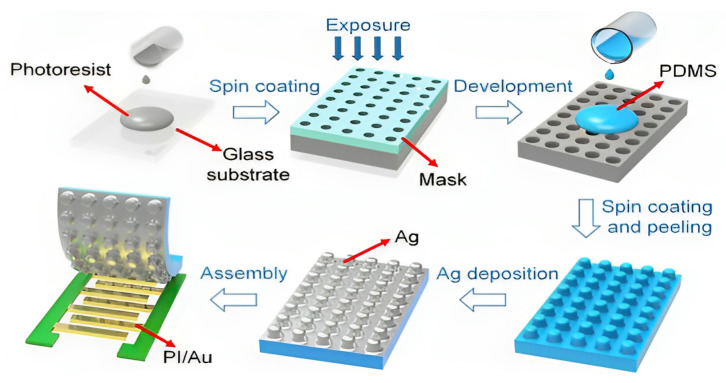
Fabrication of the rough-rough pressure sensors [49]. Copyright 2017, American Chemical Society.

It has to be said that polymer materials with special micro surface structure do have better sensing performance. Chen et al. [49] proposed and demonstrated a high-performance pressure sensor through the combination of PDMS/silver (Ag) microstructures with rough PI/gold (Au) interdigital electrodes (Figure 8), which has broad prospects in biomedicine, EH, and intelligent robot applications. The highlight of this sensor is that it uses a rough-rough configuration to achieve higher sensitivity (response time ~200 μs) compared with the flat-bottom electrodes or flat-top PDMS. It is easy to find that in rough-rough pressure sensors, the PDMS and interdigital electrode both have distinct defined conical frustum-shaped microstructures, which can provide large area, sufficient roughness, and enough elasticity. Among these merits, the eminent elastic property of PDMS/PI microstructures withstands thousands of mechanical deformation cycles. In the other research on flexible sensing, substrates or electrodes designed with pyramid microstructures [50,51,52] have also shown a similar effect. It is noteworthy that the rough microstructure is obtained by positive photoresist, and the photoresist needs to be removed in subsequent steps, making the experimental process a bit complicated. If Au is deposited on PSPI instead of PDMS, it is not required to add photoresist. Naturally, aiming to simplify the experimental process, there is no need to increase the experimental step of photoresist removal. Certainly, the premise of using PSPI is that it has benign comprehensive performance, matching the conditions in the process of sensor preparation. Apart from that, if only ordered and porous PI film is desired, it can be attained by the microemulsion droplet method [53]. The process is simple, and the layout and size of the holes can be adjusted by designing the template.

Other than the above progress (the comparison is shown in Table 2), the following works are also of great significance. The PI nanofibers prepared by Shi et al. [22] using electrospinning technology and assembled in TENG can be used at an ultra-high temperature of 250 °C with exceptional output performance. By introducing dynamic disulfide bond exchange and flexible PDMS fragments into the PI main chain, Li et al. [54] endow PI with a self-healing property, providing theoretical guidance for the production of self-healing TENG. Pang et al. [55] prepared a sandwich-like friction layer for assembling TENG by adding boron nitride nanosheets as intermediate layers between PI layers, which can retain high mechanical robustness and electrical output performance in a humid environment.

### 2.3. Sensitive Layer in RH Sensor

PI is also often used as the sensing functional layer material in sensors. For example, PI is used as the humidity-sensing material in humidity sensors owing to its sensitivity to moisture, good chemical stability, and long-term use stability in humid and hot environments, and the electrodes of the humidity sensors are usually made by inkjet printing or screen printing techniques [56]. The reason why PI can be used as the sensitive layer of an RH (relative humidity) sensor is that the dielectric constant of PI can change in the process of moisture absorption and desorption, which leads to the change of capacitance. Therefore, for the modification of PI structure, the dielectric constant is generally the parameter. However, the development of humidity sensors using PI as the sensitive material is not limited to this. As early as the end of last century, fluorinated PI and crosslinked PI have been studied for capacitive RH sensors [57]. In recent years, the emergence of fiber-optic RH sensors has given PI material a chance to show its ability. The use of new technology, the preparation of PI composites by doping, and the modification of PI structure are all effective means to enhance the performance of the RH sensor. Here are some typical examples to demonstrate.

#### 2.3.1. Sensitive Layer in Capacitive RH Sensor

**Figure 9:**
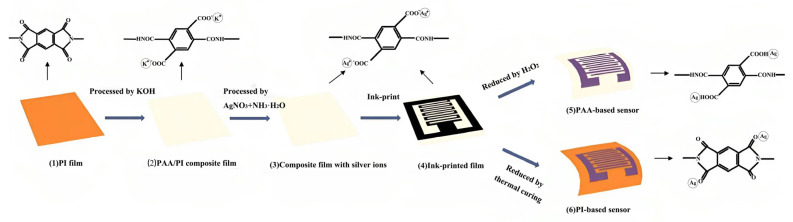
Process flow of PI capacitive humidity sensors with Ag IDEs [58]. Copyright 2015, Elsevier.

Ag is often used as the electrode of capacitive sensors and can be deposited on the PI film by ink printing. Yang et al. [58] used surface modification and ion-exchange technique to prepare PI/Ag nanocomposite films on the PI film surface and constructed two different kinds of capacitive humidity sensors with both Ag interdigital electrodes (IDE) through two different reduction processes (Figure 9). PI acts as both a sensing material and a flexible substrate in the humidity sensor. Additionally, the preparation process of Ag IDE, using simple surface modification and a patterning self-metallizing process, is also operated on PI substrate. Two kinds of humidity sensors both have excellent sensitivity at high RH standard (~70 to 90%). It is supposed that the absorbed water vapor can cause the increase of dielectric constant of polyamic acid (PAA)/PI, thereby increasing the capacitance. The results clearly show that the metallization of Ag on PI provides a viable source for future flexible applications. However, at low RH standard (~16 to 70%), the sensitivity of the humidity sensor is very low, and the dielectric constant of PI scarcely changes. The sensitivity under low RH level may be improved by introducing carboxyl or sulfonic acid groups properly into the side chain of PI. The dielectric constant parameter of PI has attracted widespread attention in the microelectronics industry. In the process of signal transmission, the material with a low dielectric constant can not only reduce the delay of signal transmission, but also improve the speed and efficiency of signal transmission. In addition to introducing fluorine-containing groups [59], a nano porous structure can be introduced to decrease the dielectric constant of PI as well.

In order to improve the sensitivity of the sensor, carbon black [60], lithium chloride, graphene [61], halloysite nanotube [62], and poly(glycidyl methacrylate) [63], and so on are doped into PI to form a composite material as a new sensitive layer.

#### 2.3.2. Sensitive Layer Based on FBG in RH Sensor

Different from the change in the dielectric constant of PI in the capacitive RH sensors, the linear volume expansion of PI under humidity conditions is the principle in the RH sensors based on FBG (fiber Bragg grating). In a harsh electromagnetic field and strongly corrosive environment, an optical humidity sensor has advantages over an electrical sensor. The RH sensor based on FBG technology [64,65,66] has been widely studied in recent years. Due to the different principles, the modification direction of the PI structure is also slightly different. Because of the large number of benzene rings and imide groups in aromatic PI, the polarity of PI is limited and results in a low expansion coefficient. Therefore, the sensitivity of RH sensors prepared by PI with small polarity is low. In contrast, if the polarity of PI is too high, the water absorption of the material will be excessive, which will lead to desorption difficulties and is not conducive to sensing. Therefore, it is necessary to increase the polarity appropriately by modifying the PI structure to improve the sensitivity of the sensor.

**Figure 10:**
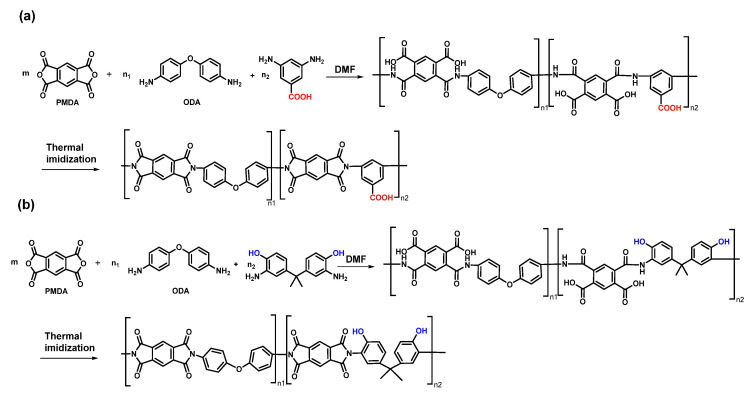
(**a**) Synthesis process of carboxyl-modified PI; (**b**) synthesis process of phenolic-hydroxyl-modified PI.

Wu et al. [67] copolymerized commercial diamine ODA and dianhydride PMDA with diamines containing phenolic hydroxyl or carboxyl groups to generate PI with phenolic hydroxyl or carboxyl groups on the backbone (Figure 10), and coated the optical fiber with PI through the impregnation method for assembling FBG sensors. The results show that the humidity sensitivity of the probe containing carboxyl PI or phenolic hydroxyl PI is 2.28 times and 1.59 times higher than that of the ordinary PI probe, respectively. However, it is still difficult to dehumidify. Studies have shown that the problem of humidity sensing delay can be solved by fluorination of PI [68]. Although the repeatability of the sensor is not good enough and the dehumidification response needs to be improved, this work still proves that the improved PI structure plays an irreplaceable role in improving the performance of the humidity sensor.

### 2.4. Insulation Layer and Dielectric Layer

PI film also has some applications as an insulating layer and dielectric layer. Here are some examples.

Du et al. [69] developed a flexible piezoresistive pressure sensor based upon an all-fiber structure, which is lightweight, ventilate, biocompatible, and highly sensitive. The device is composed of porous PVDF nanofiber film filled with conductive MXene nanosheets (MXene/PVDF) as the sensitive layer, and with magnetron sputtered Ag IDE (Ag/PVDF) as the electrode of the sensor. PI exists as an insulation layer in the middle of the sensitive layer and electrode to induce the change of contact resistance. Thanks to the presence of the insulation layer, the sensitivity of the sensor is remarkably improved (up to 1970.65 kPa^−1^ in the low-pressure range, which is about 13 times higher than that of sensors with no adjunction of insulation layer). In addition, the sensor also exhibits cycling stability (10,000 cycles), fast response time (10 ms), and fast recovery time (20 ms). More importantly, the sensor has exceedingly good air permeability and biocompatibility due to its all-fiber structure. These excellent performances provide a valuable reference for application in the sophisticated detection of human motion and pressure distribution. As an insulating layer, PI also requires low dielectric loss [70]. In recent years, as 5G communications have grown, extensive research has been conducted on the structure and preparation process of PI as an antenna material with low water uptake, low dielectric constant, and low dielectric loss [71,72,73].

**Figure 11:**
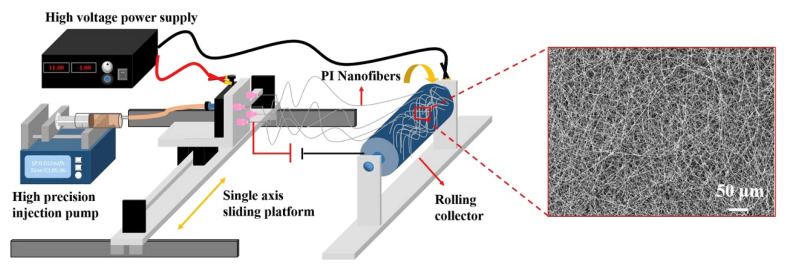
Schematic diagram of electrospun PI nanofiber preparation by four-needle electrospinning setup [74]. Copyright 2020, Elsevier.

The pressure sensor described below is also a pressure sensor using fiber, while electrospun PI fiber is used as a dielectric layer. Zhu et al. [74] used electrospun PI nanofiber membrane as the dielectric layer between the electrodes of capacitive pressure sensor to achieve superior sensitivity (2.204 kPa^−1^ in 3.5–4.1 Pa and 0.721 kPa^−1^ in 4.1–13.9 Pa), wide scale range (0–1.388 MPa), low detection limit (3.5 Pa), and eminent cycle stability (>10,000 cycles). Generally, the dielectric materials used in capacitive pressure sensors should be easy to compress and have small Young’s modulus, such as PDMS, silicone rubber, and so on. In this respect, PI is not suitable as a dielectric layer. However, the performance of a dielectric layer can be optimized by using a multi-pore structure. Void structure is often used in polymer to improve the elastic deformation ability of the dielectric layer, so as to increase the sensitivity of sensors. The fiber membrane is increasingly favored by pressure sensors because of its ultra-high porosity. When atmospheric pressure is applied, the membrane can be easily compressed, which reduces the electrode distance of the capacitive pressure sensors, and the equivalent dielectric coefficient increases, so that the total capacitance increases rapidly. In addition, PI has good stability, so the electrospun PI fiber membrane has been used as a dielectric layer by a four-needle electrospinning setup (Figure 11), and the performance of the sensor used as a dielectric layer is compared with PDMS. The results show that the electrospun PI fiber membrane has better sensitivity and a lower detection limit, which can effectively improve the performance of the capacitive pressure sensor.

### 2.5. Sacrificial Layer

The selection of sacrificial layer materials for sensors is one of the most important factors to consider. When metals such as aluminum (Al) or tin are inevitably used as electrodes in sensors, it is difficult to directly make electrodes due to the poor selectivity of metals for hydrogen fluoride (HF) etching. In this case, PI can be selected as the sacrificial layer [75,76,77]. Compared with the traditional oxidation sacrificial layer, the oxygen plasma ashing etching selectivity of PI is superior.

**Figure 12:**
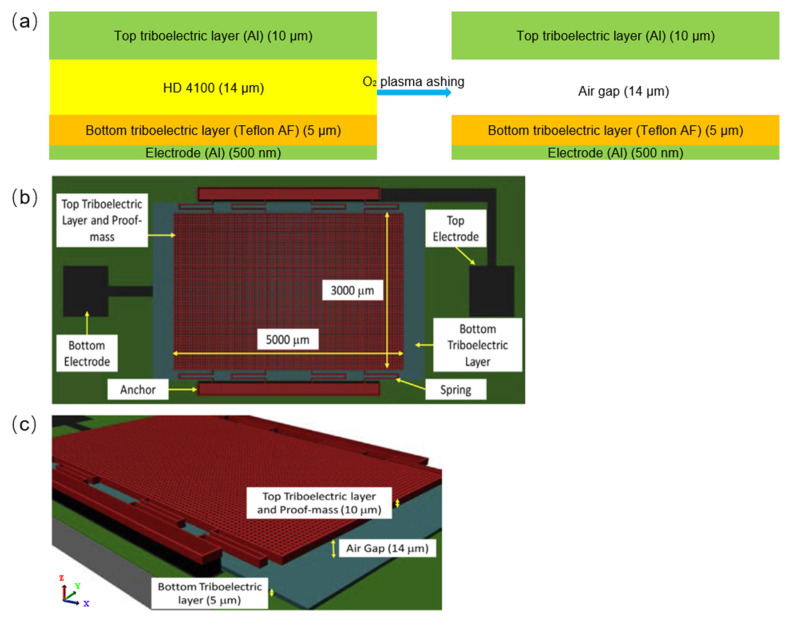
(**a**) Schematic diagram of introducing air gap; (**b**) top view of structural model of TEH; (**c**) tilted view of structural model of TEH [78]. Copyright 2020, Elsevier.

**Figure 13:**
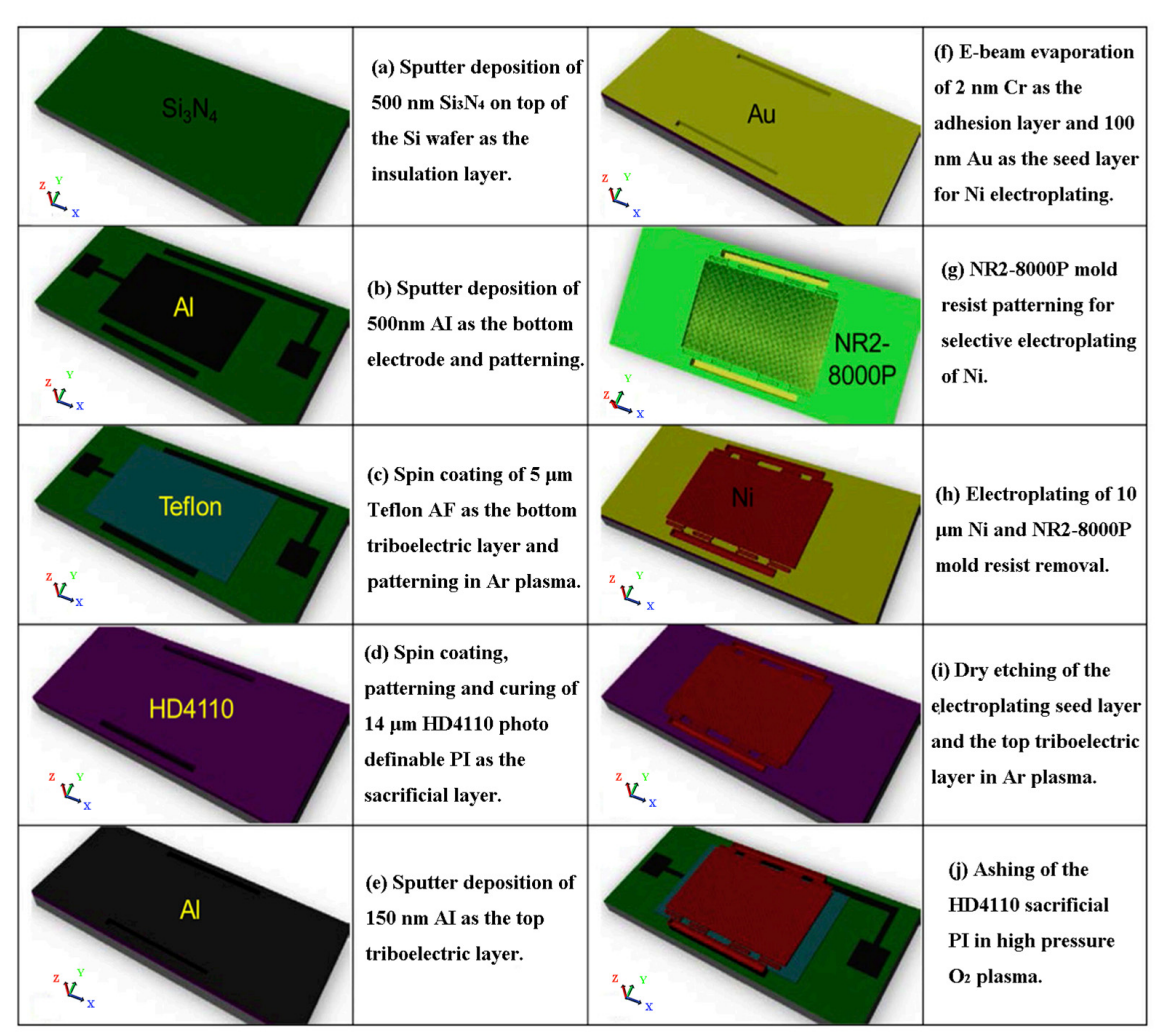
Complete and detailed manufacturing process for TEH [78]. Copyright 2020, Elsevier.

Hamid et al. [78] presented a new triboelectric EH and sensing system, which can be reduced to the size of microelectromechanical systems (MEMS). In general, there are four working modes of TENGs, including vertical contact separation mode, horizontal sliding mode, single electrode mode, and independent layer mode [79,80,81]. The vertical contact separation mode is adopted here, and the system uses eight serpentine springs to realize the suspension of the top triboelectric layer (Figure 12b,c). When the system is in a sufficiently strong periodic vertical external vibration, the air gap can reach zero, and periodic contact and separation can occur between the triboelectric layers to achieve current output. In order to reduce the adverse effects of the scale reduction, it is necessary to increase the air gap between two triboelectric layers by introducing and removing HD 4100 (a kind of n-PSPI) (Figure 12a). HD 4100 undergoes two steps of curing at 200 °C and 365 °C for 30 min and 1 h, respectively, to form a 14 μm thick film. The designed structure of the triboelectric EH is optimized to obtain the maximum average power and power density, while guaranteeing the robustness of the structure and achieving high operating frequency and wide bandwidth. The complete and detailed manufacturing process of the triboelectric energy harvester (TEH) is shown in Figure 13. Throughout the manufacturing process, HD 4100 acts as a sacrificial layer for the triboelectric EH, which is primarily used for patterning of the top triboelectric layer and finally removed by oxygen plasma ashing technique to obtain an air gap. The novel triboelectric EH system is able to be integrated into the self-powered sensors and gain specific applications (e.g., automobile industry, actuator systems in airplane, prosthetic systems, and micro-robotic systems).

In addition to using PI as a sacrificial layer, pattern transfer can also be achieved by selecting appropriate flexible substrates through clean and environmentally friendly contact printing (i.e., no additional organic solvents and chemicals required) [13] without using a sacrificial layer.

### 2.6. Coating Material

PI is widely used as a packaging material in the field of semiconductor chips and separation devices. In addition to the protective barrier, PI is often doped into a sensitive layer as a composite material to improve sensing performance [82]. Besides the FBG RH sensor based on PI coating [83] mentioned in Section 2.3.2, PI is also applied in other sensing fields as an optical fiber coating [84,85]. PI coating can not only improve the mechanical properties and heat resistance of the optical fiber sensor, but also effectively protect the optical fiber, and even improve the sensitivity of the sensor [84]. For an oil downhole monitoring application, the PI-coated fiberoptic distributed temperature sensor can work at 300 °C [86].

**Figure 14:**
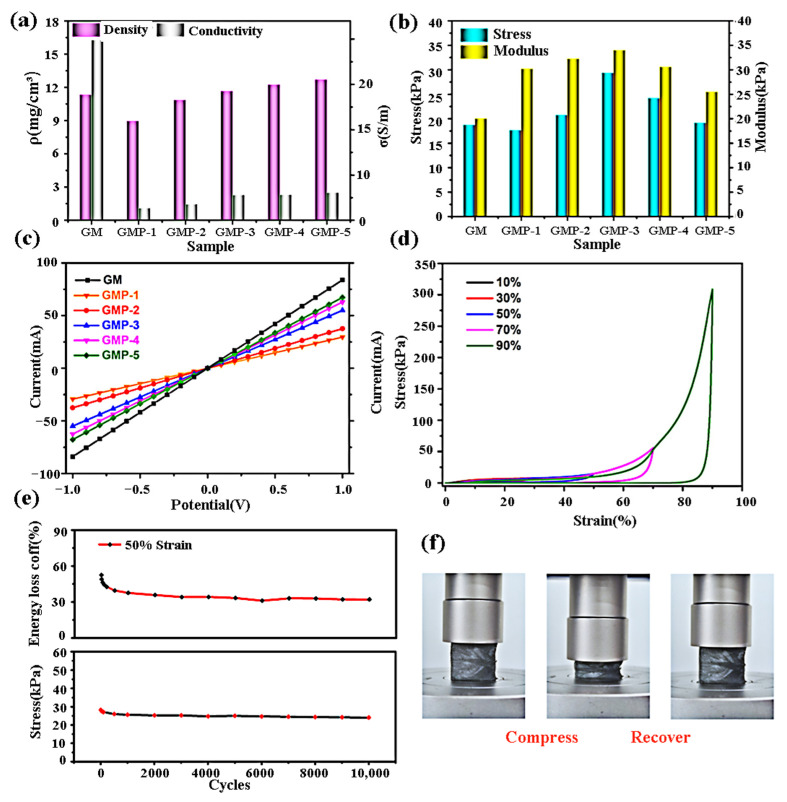
(**a**) Density and conductivity of various aerogel samples; (**b**) stress and modulus of various aerogel samples; (**c**) current–potential curves of various aerogel samples; (**d**) stress curves of the GMP-3 composite aerogel at various strains; (**e**) stress−strain and energy loss curves of the GMP-3 composite aerogel under various cycles (at 50% strain); (**f**) schematic diagram of cyclic compression [87]. Copyright 2022, American Chemical Society.

Graphene is often used in piezoresistive sensors because of its excellent electrical and mechanical properties, but its preparation difficulty and stability limit its development. A relatively simple preparation process is using PI as the carbon source to prepare graphene, which will be discussed in Section 3. Here, an effective strategy to improve the stability of graphene is to compound it with polymer. Yang et al. [87] assembled a highly sensitive piezoresistive sensor based on a porous composite aerogel of reduced graphene oxide (rGO)/MXene/PI (GMP (G:rGO, M:MXene, P:PI), the name of porous composite aerogel) that can be used to monitor tiny and complicated human motions (e.g., breathing, pulsing, finger bending, etc.). PI acts as a binder and reinforcing agent for GO and MXene sheets to prepare robust GMP composite aerogels. MXene sheet is pulled to the outer surface of the rGO sheet through hydrogen bond and strong adhesion of PAA to achieve a high-quality, continuous three-dimensional (3D) ternary aerogel. Several kinds of GMP aerogels with various MXene mass ratios to GO are synthesized for performance comparison. Among them, the GM aerogel sample is contrasted with no addition of PAA. The experimental results are shown in Figure 14. This proves that the introduction of the PI precursors can transform the brittle rGO/MXene aerogel into a composite aerogel with better elasticity and flexibility. The synergistic effect of rGO/MXene enhanced by PI provides GMP composite aerogel with prime electrical and mechanical properties. Furthermore, it has superior heat insulation performance, low and high temperature resistance, and flame retardance. It is remarkable that the GMP aerogel is obtained by a directional freezing procedure to realize low density (8.97–12.71 mg/cm^3^). PIs are required to have low density [88] if they want to be applied to human skin. Yao et al. [89] provided a freezing-extraction/vacuum-drying method for PI fibrous aerogel with low density (≤52.8 mg/cm^3^), robustness, and fatigue resistance.

**Figure 15:**
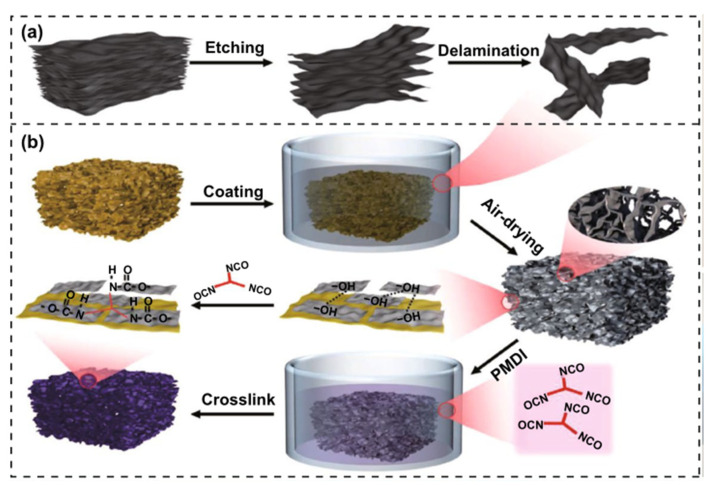
(**a**) Fabrication process of MXene sheets; (**b**) preparation process of C-MXene@PI composite foam [90]. Copyright 2022, Springer Nature.

For MXene, similar to graphene, an analogous strategy can also be adopted. Zeng et al. [90] manufactured flexible, lightweight, and robust cross-linked transition metal carbide (Ti_3_C_2_ MXene) for coating PI (C-MXene@PI) porous composites through scalable dip coating and chemical crosslinking methods. The preparation principle is that MXene can adhere well to the PI skeleton through the strong hydrogen bond interaction between PI and MXene nanosheets; thereby, MXene-coated PI composite foams are successfully prepared. In addition, C-MXene@PI is obtained by the further chemical crosslinking agent poly ((phenyl isocyanate) -co-formaldehyde) (PMDI) (Figure 15). The composite foam successfully achieves oxidation resistance, hydrophobicity, and extreme temperature stability. Moreover, the composite foam attaches well to the human body to detect its electromechanical sensing performance, proving that it is reliable and sensitive as a wearable sensor to detect human movement, and it has potential to realize the application of PI on human skin. The robust and porous PI bracket can endow the composite foams with excellent flexibility, low density, and extreme temperature resistance properties.

## 3. Application of PI Graphitization to Graphene in Sensors

PI film can be converted into carbon nanomaterials (e.g., carbon nanotubes (CNTs), graphene) via graphitization, and it is commonly regarded as a relevant pyrolysis precursor of graphitized films used in diverse electrodes and batteries. Carbon nanomaterials have great advantages in sensors because of their excellent conductivity, chemical robustness, and sensitivity to a wide range of analytes, especially in electrochemical sensing that requires high sensitivity and selectivity, fast response, and low cost [91,92,93]. Here, in addition to the application of PI in sensors by traditional high-temperature graphitization, LIG based on PI is also introduced. 

### 3.1. High-Temperature Graphitization of PI

**Figure 16:**
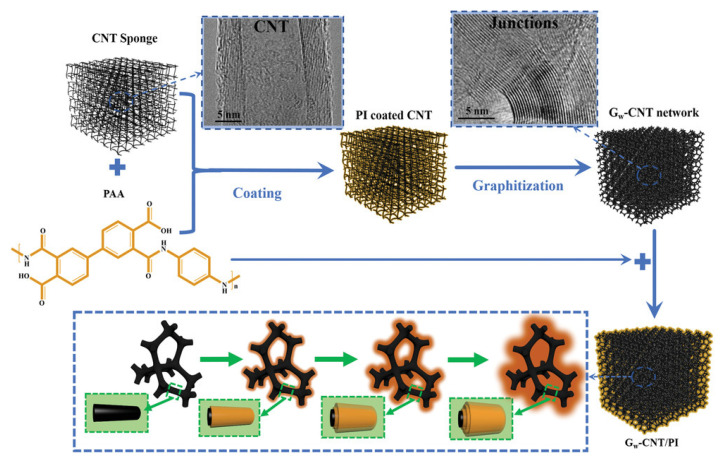
Fabrication process of 3D elastic G_w_-CNT/PI nanocomposites [94]. Copyright 2019, John Wiley and Sons.

In 2019, Zhang et al. [94] presented a 3D elastic graphene-crosslinked carbon nanotube sponge/PI (G_w_-CNT/PI) composite. Based on the experiments and theoretical simulations, the G_w_-CNT/PI-5 composite with the highest electrical sensitivity (sensitivity, η = 973% at 9.6% strain) is promising as an alternative material for flexible piezoresistive sensors [2]. The high sensitivity is attributed to a combination of relatively low conductivity and suitable compression strain. Crosslinked G_w_-CNT hybrid networks give PI high modulus with controllable compression deformability and elasticity, making it suitable for use in flexible electronics. From Figure 16, the 3D interconnected G_w_-CNT hybrid network structure is prepared by coating a PI layer and then performing graphitization, and the final PI nanocomposites (G_w_-CNT/PI) are obtained by coating the G_w_-CNT frame with PI layer by layer. The elasticity, thermal conduction, and electronic conduction of the G_w_-CNT/PI nanocomposites are influenced by the composite microstructure, which can be facilitated by the layer-by-layer coating of PI. Furthermore, the strong interactions between PI and G_w_-CNT enable uniform synthesis, and the structural integrity can be retained by avoiding network expansion or contraction during the solution process. The introduction of an interconnected cross-linked structure can improve the number of cycles of the G_w_-CNT/PI composites. However, the preparation process has an inherent defect, that is, the graphite structure changes when the heat temperature of carbonization and graphitization are between 1000 °C (C_w_-CNT) and 3000 °C (G_w_-CNT).

### 3.2. LIG Based on PI

Generally, synthesis methods of porous graphene require high-temperature treatment [42] or a multi-step chemical synthesis route [95]. Although great efforts have been paid to achieve low-temperature or chemical-free processing of high-quality graphene, low-cost synthetic methods for directly manufacturing graphene sheets on the substrate are still rare. LIG [96,97,98] is a recently developed method for the direct formation of graphene from carbon-rich materials. Lin et al. [99] demonstrated that LIG exhibits high electrical conductivity through experiments and found that the mechanism of laser graphitization in polymers is closely related to the structural features present in the repeating units, such as aromatic and imide repeating units. PI, as a carbon-rich material containing aromatic and imide repeating units, can be used to form LIG by using a CO_2_ infrared laser under environmental condition. Tao et al. [100] developed a wearable, one-step, and low-cost LIG artificial throat using direct laser writing of PI. The LIG artificial throat can produce sound with controlled volume and frequency by detecting various types of simulated hum. It is experimentally found that PIs of different thicknesses show differences in recognition due to different resistance change.

**Figure 17:**
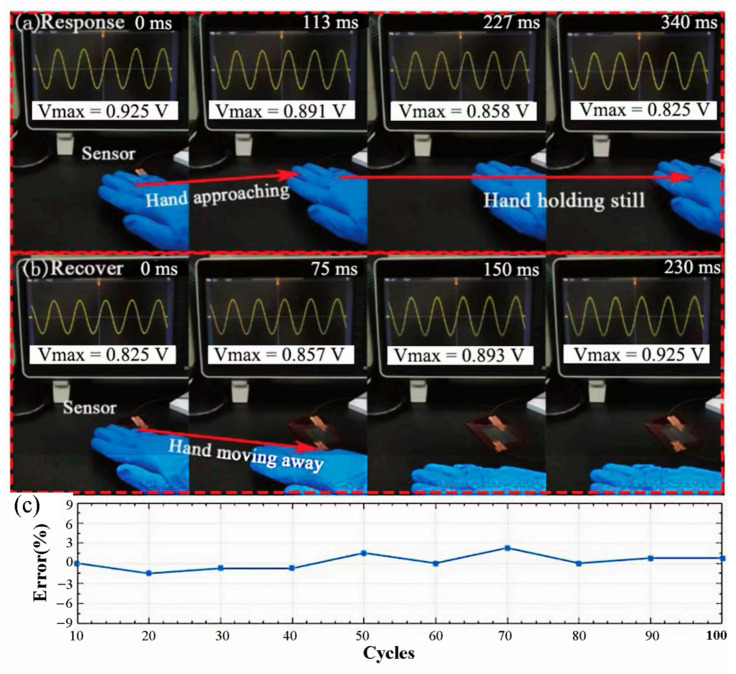
(**a**) Response/recovery when approaching the sensor; (**b**) response/recovery when moving away from the sensor; (**c**) the response variation diagram of the sensor when frequently running for 100 cycles [101]. Copyright 2019, John Wiley and Sons.

The laser power has a great influence on the structure and properties of LIG. Only when the power reaches a certain amount (≥2.4 W) can the polymer film be carbonized effectively [99]. Chen et al. [101] developed a system for preparing high-quality graphene directly from PI films at room temperature and atmospheric pressure using a picosecond UV laser and explored and determined the effects of the laser processing parameters on the quality of LIG. After optimization, they fabricated a high-sensitivity proximity sensor to prove the promising application of LIG. Figure 17 shows the response and recovery when a human hand approaches or moves away from sensors, respectively. Meanwhile, it also shows that the response voltage varies little when the hand is constantly monitored for 100 cycles. The above results indicate that the LIG proximity sensor has decent sensitivity and stability and is able to detect the approaching objects promptly. Although the sensitivity of this sensor needs to be further improved, it still exhibits the broad application potential of the UV LIG technique.

It is notable that due to the convenient processability of polymers, graphene hybrid with the required properties can be obtained by changing the polymer composition. For example, Peng et al. [102] prepared boron-doped porous graphene in ambient air by means of a facile laser induction process from boric acid comprising PI sheets, which can be utilized as an useful active material for a flexible in-plane micro-supercapacitor. With boron doping, the electrochemical property of boron-doped LIG is significantly improved. Furthermore, the transformation of PAA to PI is proven to be vital for the successful formation of LIG with both a good electrochemical property and high quality. LIG based on PI has more application in biosensors [103,104] and wearable sensors [105]. In 2022, Li et al. [104] presented a neurotransmitter sensor for the brain and gut that resembles tissue. A polymer precursor solution including PAA mixed with a metalloporphyrin is casted as a film on a PI substrate and then annealed in air in order to form the PI film. The film surface, through laser carbonization, can generate the graphene network. The sensitivity and selectivity of the sensor can be enhanced by mixing transition metal nanoparticles, which can promote molecular absorption and electron transfer. Significantly, PI nanoparticles formed by laser carbonization are embedded in elastomers and can exist in animal and human bodies. This provides a great value in achieving the biocompatibility of PI.

## 4. Challenges and Perspectives Analysis

Although there have been many studies about PI, many review papers mainly reported the usage of PI as a substrate in flexible-substrate electronics. PI film (Kapton) has been recognized as a flexible substrate due to its chemical stability, heat resistance, and good mechanical properties. However, as a traditional organic polymer, past studies have focused more on how to improve the mechanical and thermal properties of PI to realize its applications in aeronautics, aerospace, and the military industry [106,107]. However, with the development of 5G communication, sensing technology, and flexible display, more attention has been paid to the dielectric and optical properties of PI in order to realize the application of PI in the above fields. As different layers, PI materials have different functions, so the required properties are also different. For example, as the sensitive layer of an RH sensor, PI needs a low dielectric constant to obtain high sensitivity. However, as a triboelectric layer, PI needs a high dielectric constant to achieve high power output. It is difficult to predict the dielectric property of PI by simple structure–property relation algorithms. PI, as a dielectric layer or insulating layer, requires low dielectric loss. However, the relation between dielectric loss and the structure of the polymer is difficult to express. Due to the trade-off effect of material properties, it is difficult to obtain a relatively accurate result by using the local optimization algorithm and global optimization algorithm. It is also unrealistic to test the performance of PIs one by one, although this can provide relatively accurate results. If a small amount of experimental sample testing and an existing database are used, it is the most ideal situation to achieve bidirectional input and output of material structure and performance (i.e., input performance data and output structure reference, input structure data and output performance prediction). 

As a significant branch of artificial intelligence (AI), machine learning (ML) is a strong tool for interpreting sensor data easily with an outstanding advantage of effectively handling multi-dimensional and multi-faceted data, which is very complicated [108]. Through the combination of materials and AI, it is expected to infer the performance of polymers with unknown structures from existing structures through ML assisted by specific algorithms; this could even give the corresponding material structure and composition by providing only the required material properties. It is encouraging that researchers have tried to establish high-throughput screening through ML and made progress at glass transition temperature and cut-off wavelength of PI recently [109,110]. With the emergence of ML and deep learning, data-driven chemical informatics research has made new progress. Through the input of chemical and physical properties, the composition, structure, and performance can be realized through ML [111,112].

## 5. Conclusions

This review mainly summarizes the recent study advances regarding the applications of PIs in flexible sensors, including PI aerogel, electrospun PI fiber membrane, modified PI film, PSPI, and so on as flexible substrate, friction layer, sensitive layer, sacrificial layer, dielectric layer, and coating. The combination of PI with advanced sensing technologies based on TENG and FBG has demonstrated the importance of PI in the sensing field. In particular, the application of PI graphitization to graphene as a convenient processing technology in sensors is introduced with a simple laser-induced method. Although PI material has many merits, there are still problems in the practical application of flexible sensors. Based on these problems, future efforts should be made in designing new structures of PI with high performance and developing new processing technology; at the same time, ML can be integrated to accelerate the development of innovative processing technology and the establishment of a database dedicated to PI. It has to be emphasized that the performance requirements for PI vary between different sensors. For example, (a) in wearable devices, PI requires ultra-light weight, low water absorption, and high transparency, which can be achieved by introducing silicon-oxygen bond and non-coplanar ring structures, and so on, in the main or branch chains of PI; while (b) in biosensing and environmental monitoring, the use of electrospinning technology can directly produce PI nanofibers, making PI with the necessary property of high air permeability. In order to truly realize the large-scale application of flexible sensing, it is essential to constantly not only improve the properties of polymer materials like PI, but also combine them with ML to achieve more breakthroughs in developing processing technologies and setting up databases about various polymer materials.

## Figures and Tables

**Table 1:** Sensing performance comparison of PI and membrane filter substrate [27].

	Sensitivity			Response Time/ms	Recovery Time/ms
	In Low Pressure/kPa^−1^	In Middle Pressure/kPa^−1^	In High Pressure/kPa^−1^		
PI	281.54	509.78	66.68	67.8	44.8
Membrane filter	99.8	408.4	23.4	68.4	46.5

**Table 2:** Comparison and analysis of different PI-based TENGs.

Name	Parameters	Advantages	Disadvantages	Ref.
50% open-cell content PI aerogel	V_oc_: 40 V I_sc_: 5 μA P_t max_: 47 μW	Lightweight, efficient	The process is complex and uneconomical	[20,21]
Fluorinated PI (6FDA-TFDB)	V_oc_: 30 V I_sc_: 0.4 μA Charge density: 24.82 μC/m^2^	High transparency, durability	Low power output, structural requirements	[44]
Md-PI (md-PI_95)	V_oc_: 122.20 V I_sc_: 4.4 μA Output power: 1.42 W/m^2^ Charge density: 58.4 μC/m^2^	Superior electrical output, durability, and thermal stability	Strict requirements for PI solubility	[47]

## Data Availability

Not applicable.
